# Peptide Functionalization of Gold Nanoparticles for the Detection of Carcinoembryonic Antigen in Blood Plasma via SPR-Based Biosensor

**DOI:** 10.3389/fchem.2019.00040

**Published:** 2019-02-04

**Authors:** Maria Laura Ermini, Xue Chadtová Song, Tomáš Špringer, Jiří Homola

**Affiliations:** Institute of Photonics and Electronics, Czech Academy of Sciences, Prague, Czechia

**Keywords:** gold nanoparticles, SPR, functionalization, ζ-potential, peptide, immuno-assay, biosensor, blood

## Abstract

Nanoparticles functionalized with specific biological recognition molecules play a major role for sensor response enhancement in surface plasmon resonance (SPR) based biosensors. The functionalization procedure of such nanoparticles is crucial, since it influences their interactions with the environment and determines their applicability to biomolecular detection in complex matrices. In this work we show how the ζ-potential (Zpot) of bio-functionalized gold spherical NPs (Bio-NPs) is related to the SPR sensor response enhancement of an immune-sandwich-assay for the detection of the carcinoembryonic antigen (CEA), a cancer marker for colorectal carcinomas. In particular, we prepare bio-functional nanoparticles by varying the amount of peptide (either streptavidin or antibody against CEA) bound on their surface. Specific and non-specific sensor responses, reproducibility, and colloidal stability of those bio-functional nanoparticles are measured via SPR and compared to ζ-potential values. Those parameters are first measured in buffer solution, then measured again when the surface of the biosensor is exposed to blood plasma, and finally when the nanoparticles are immersed in blood plasma and flowed overnight on the biosensor. We found that ζ-potential values can guide the design of bio-functional NPs with improved binding efficiency and reduced non-specific sensor response, suitable reproducibility and colloidal stability, even in complex matrixes like blood plasma.

## Introduction

Bio-functional gold nanoparticles (Bio-NPs) have gained significant popularity in recent years, primarily for their suitability in a multitude of biomedical and bioanalytical applications. They are widely applied *in vitro* for the detection of clinically relevant molecules (Farka et al., 2017; Mittal et al., 2017) and *in vivo* as both a diagnostic tool and a therapeutic agent (Barkat et al., 2001; Galanzha et al., 2009; Nie et al., 2014; Jo et al., 2015; Rejeeth and Kannan, 2016; Rizk et al., 2016; Falagan-Lotsch et al., 2017; Lather et al., 2018).

Functionalized *ad hoc*, nanoparticles (NPs) can be useful *in vivo* for targeting cancer (Rejeeth and Kannan, 2016) or likewise for cancer therapy (Nie et al., 2014). In clinical biosensing, NPs are usually functionalized prior to the measurements with a specific receptor for the target analyte. When utilized in biosensing, they selectively react with a target molecule, thus enhancing the detection sensor response (Graphical Abstract). This approach is widely used in SPR biosensors (Shen et al., 2014; Wang et al., 2015), as well as other biosensors based on different techniques (Farka et al., 2017; Zhang et al., 2018); for example, in colorimetric biosensors the aggregation of colloidal NPs is directly related to the presence of the analyte (Wang et al., 2015). In SPR biosensing, Bio-NPs usually act as a sensor response enhancing protagonists, functionalized with a ligand and frequently applied in sandwich assays (Shen et al., 2014). In this type of experiment, Bio-NPs carry a specific ligand for the target, for example an antibody (de la Escosura-Muniz et al., 2010; Viswambari Devi et al., 2015; Wang et al., 2015), that provides specific sensor response enhancement. In other more complex approaches, a biotinylated secondary antibody detects the target molecule in a sandwich assay, after which the Bio-NPs enhance the sensor response of the target-specific recognition, through a biotin-streptavidin interaction (Haes and Van Duyne, 2002; Mitchell et al., 2005; Kajiura et al., 2009; Martinez-Perdiguero et al., 2014; Špringer et al., 2014).

**GRAPHICAL ABSTRACT:**
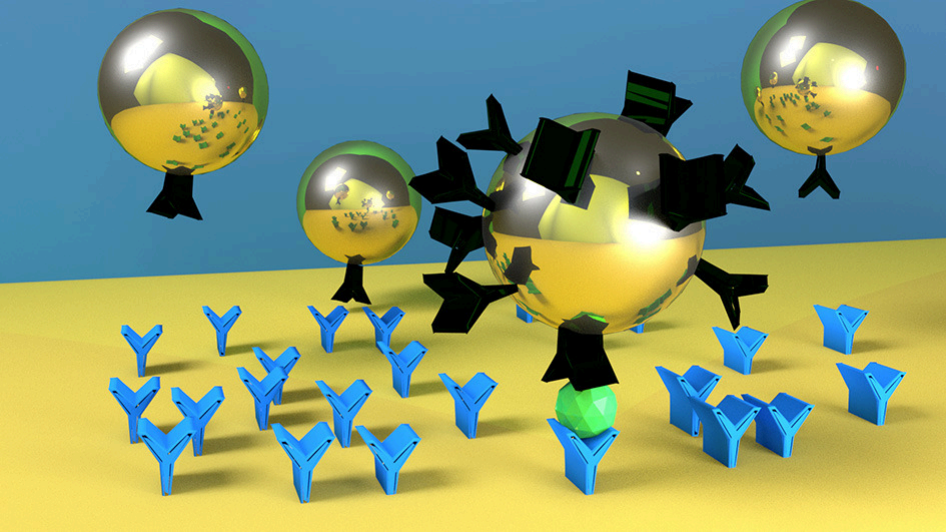
Nanoparticles with higher number of ligands on their surface (here represented as black molecule) are faster and more specific in detecting the target molecule (green one). Blue molecules represent the antibodies on the SPR biosensor surface.

Different strategies are used to attach the ligand on the NPs. The most used strategy consists in creating a thiol self-assembled monolayer (SAM) for the amino-coupling reaction with the functional groups of the ligand (Liu et al., 2007; Rausch et al., 2010; Sanz et al., 2012; Zhang et al., 2014). Using materials such as polyethylene glycol (PEG) in the SAM, it is possible to reduce the corona effect on NPs in biological samples (Sacchetti et al., 2013; Dai et al., 2014; Liu et al., 2017). It associates with water molecules, creating a barrier on the NPs surface that blocks the adsorption of other proteins. In addition, zwitterionic material (Ou et al., 2018) have been recently used as well as polymers (Cheng et al., 2018; Chortarea et al., 2018).

Several approaches have been reported in the literature for efficiently producing Bio-NPs that are stable and specific, even in biological samples. For these applications Bio-NPs must be endowed with several characteristics for providing a successful, fast, and specific detection. The fundamental characteristics to be considered for optimized detection are affinity, non-specific interactions, and reproducibility. Hence, in addition to the selection of the functional specific biomolecule, the design of the NP functionalization also maintains crucial importance.

Here we report how the performance of Bio-NPs—measured in terms of specificity, non-specific sensor response, and reproducibility—depend on the Bio-NP surface design. We use the ζ-potential (Zpot) as a predictive parameter optimized sensor response enhancement in a SPR biosensor, both in buffer and in blood plasma. We first evaluate specificity, non-specific sensor response and reproducibility of the SPR sensor response enhancement (SPR sensor response), regarding the detection of a model cancer marker in buffer. To extend the range of possible applications, those Bio-NPs are used on the same SPR assay after treating the surface with blood plasma, exposing them to the same situation as that for detection of the same analyte from a blood plasma sample. We furthermore report SPR sensor responses obtained when the Bio-NPs are immersed in blood plasma; in these tests the SPR sensor acts as a tool for testing Bio-NPs properties under *in vivo*-like conditions (NPs immersed in plasma for several hours), mimicking the stress that they might encounter during *in vivo* application.

## Materials and Methods

### Reagents And Solutions

HAuCl_4_. 3H_2_O (99%) and trisodium citrate (99%) are purchased by Sigma-Aldrich. Sodium acetate buffer solution 3M, pH 5.2 (25°C), KH_2_PO_4_, Na_2_HPO_4_, KCl, NaCl, ethanolamine, bovine serum albumin (BSA), streptavidin, and glutaraldehyde are purchased in molecular biology grade or higher from Sigma-Aldrich, USA. SA_10_ solution is obtained by diluting the commercial solution to 10 mM in sodium acetate, pH 5 at 25°C. The phosphate buffer (PBS) consisted of 1.4 mM KH_2_PO_4_, 8 mM Na_2_HPO_4_, 2.7 mM KCl, 137 mM NaCl, pH 7.4 at 25°C. PBS_NaCl_ is obtained, raising the NaCl concentration of the phosphate buffer to 750 mM. PBS_BSA_ buffer is prepared by adding BSA to PBS to reach a concentration of 250 μg/mL. N-Hydroxysuccinimide (NHS) and 1-ethyl-3-(3-dimethylaminopropyl) carbodiimide hydrochloride (EDC) are purchased from GE Healthcare, USA. Primary IgG1-type antibody (Ab_1_) against CEA, secondary IgG1-type antibody (Ab_2_) against CEA, biotinylated secondary IgG1-type antibody against CEA (Ab_2_B), and CEA are all purchased from Fitzgerald, USA. Carboxy-terminated (HS-(CH_2_)_11_-(O(CH_2_)_2_)_6_-OCH_2_-COOH) and hydroxyl-terminated (HS–(CH_2_)_11_-(O(CH_2_)_2_)_4_-OH) thiols are purchased from Prochimia, Poland. Ethanol for spectroscopy (purity 99.9% or greater) is purchased from Merck, USA. All buffers are prepared using deionized water (18 MΩ/cm resistivity, Direct-Q from Millipore). Normal human plasma (mixed/pooled gender) in sodium citrate was purchased from Biochemed Services and stored at−80°C until use.

### NPs Synthesis

Thirty nano meter gold spherical NPs are synthetized using a seeded growth strategy via HAuCl_4_ reduction with trisodium citrate as reported by Bastús et al. (2011). Briefly, Au seeds are produced by adding 1 mL of HAuCl_4_ solution (25 mM) to a boiling aqueous solution of trisodium citrate (2.2 mM). The solution is allowed to cool to 90°C, after which 1 mL of a trisodium citrate solution (60 mM) and 1 mL of a HAuCl_4_ solution (25 mM) are added in sequence. These additions are repeated another three times after the first 30 min. The resulting solution contains about 7.6 × 10^11^ NPs/mL of 30 nm ± 6 nm in diameter (determined from at least 10 SEM pictures).

### NPs Functionalization

Two kinds of NP-surface design have been studied in this paper: covalently bound streptavidin (S-NPs) and covalently bound secondary antibody (Ab_2_-NPs).

#### Thiol Modification of NPs

For functionalization of bare NPs with COOH-thiols, a mixture of 18 mL of 682 pM bare NPs (maximum peak of absorbance: 0.2, 4 × 1011 NPs/ml) and 180 μL of 10 mM COOH-thiols (dissolved in 100% spectroscopic ethanol) is sonicated in a water bath (50°C) for 45 min and subsequently shaken at room temperature for 3 h.

#### Washing Off Thiols

Unreacted COOH-thiols from the solution are removed in five washing cycles. In each cycle, twelve tubes with 1.5 mL thiolated NPs is centrifuged (9,500 *g*, 10 min), after which the supernatant is discarded and the pellet is dissolved in Q+NaOH solution (= 25 mL Q water + 30 mL 100 mM NaOH). After the last washing cycle only 0.25 mL Q + NaOH solution is added to concentrate thiolated NPs in a solution.

#### Activation of COOH Thiols and Incubation With S or Ab_2_

To activate carboxy-groups, 250 μL thiolated NPs at a concentration of 4 nM is mixed with 120 μl NHS/EDC solution (1 mM NHS and 5 mM EDC in Q water). This mixture is shaken (for 2 min), centrifuged (9,500 g for 3 min) and then the supernatant is removed (total activation time is 5 min). The pellet is dissolved in 500 μL protein solution (S or Ab_2_) and the mixture is shaken for 1 h. Three different streptavidin solutions are prepared. For a streptavidin:NP molar ratio of 20:1, we mixed 1.2 μg streptavidin in 1.2 μL SA_10_ with 98.8 μL PBS and 400 μL Q water; for a molar ratio of 50:1, we mixed 3 μg streptavidin in 3 μL SA_10_ with 97 μL PBS and 400 μL Q water; and for a molar ratio of 200:1, we mixed 12 μg streptavidin in 12 μL SA_10_ with 88 μL PBS and 400 μL Q water. Three different Ab_2_ solutions were then prepared. For a Ab_2_:NP molar ratio of 20:1, we mixed 3 μg Ab_2_ in 3 μL Q water with 75 μL PBS and 422 μL Q water; for molar ratio of 50:1, we mixed 7.5 μg streptavidin in 7.5 μL Q water with 75 μL PBS and 417.5 μL Q water; and for a molar ratio of 200:1, we mixed 30 μg Ab_2_ in 30 μL Q water with 75 μL PBS and 395 μL Q water. After incubation with streptavidin, we added 50 μL 1M ethanolamine, pH 8, for 5 min to deactivate all non-reacted esters.

#### Washing Off Unbound Molecules

Free proteins from the solution are removed in 6 washing cycles (9,500 g, 10 min), where supernatant is removed from each tube and the pellet is dissolved in 500 μL Q+NaOH solution. The cross-linked NPs are subsequently removed from the solution in 2 “soft” centrifugation cycles: solutions with NPs is centrifuged (210 g, 5 min), supernatant is kept, and the pellet is removed. The resulted solution is stored in a fridge.

### NP Characterization

**Figure 1:**
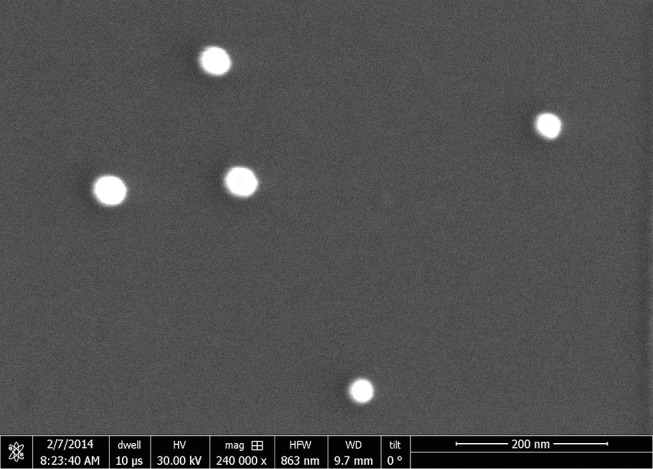
SEM image of citrate covered NPs deposited on a silicon substrate (image taken with e_LiNE plus, Raith, Germany).

The shape and size of the NPs prior to functionalization (Figure 1) is confirmed by SEM images (e_LiNE plus system produced by Raith, Germany). The ζ-potential of ligand-conjugated NPs, immersed in a NaOH water solution (pH = 8), is measured using a ZetaPals instrument produced by Brookhaven Instruments Corporation, USA.

### SPR Biosensor

For the measurements in buffer we use a four-channel SPR sensor with wavelength modulation (Plasmon IV) (Vaisocherová et al., 2006) using dispersionless microfluidics developed at the Institute of Photonics and Electronics, Prague (Špringer et al., 2010a,b). The chip is prepared as reported by Špringer et al. (2014). For each experiment a single glass chip covered with thin gold layer is used and applied for measurement of two different NPs (two channels for detection and two as a reference).

Prior to each experiment, the SPR chips are incubated with a 3:7 mixture of carboxylated thiols 0.2 M (HS-(CH_2_)_11_-(O(CH_2_)_2_)_6_-OCH_2_-COOH and HS–(CH_2_)_11_-(O(CH_2_)_2_)_4_-OH) for 10 min at 40°C. Chips are rinsed with ethanol and deionized water, dried with nitrogen, and mounted to the SPR sensor. Primary antibody is immobilized on the surface [10 μg/mL (67 nM) in SA_10_ for 15 min, 20 μL/min] after surface carboxyl group activation (0.5 M NHS/0.1 M EDC in SA_10_ for 10 min, 3 μL/min). Once maximum coverage is reached, the surface is washed with PBS_NaCl_ for 5 min (20 μL/min) to remove the non-covalently bound Ab_1_, and with 1 mM aqueous ethanolamine (pH 8 at 25°C) for 5 min (20 μL/min) to deactivate all unreacted carboxyl groups.

### SPR Experiments

The model assays used in this work are two variations of a sandwich-assay for the detection of carcinoembryonic antigen (CEA), a cancer marker related to colorectal carcinomas (Špringer et al., 2014). In all experiments, CEA (100 ng/mL (0.5 nM) in PBS_BSA_) is pumped for 2 min (20 μL/min) only through the detection channels, until a SPR sensor response of 0.1 nm is reached.

**Figure 2:**
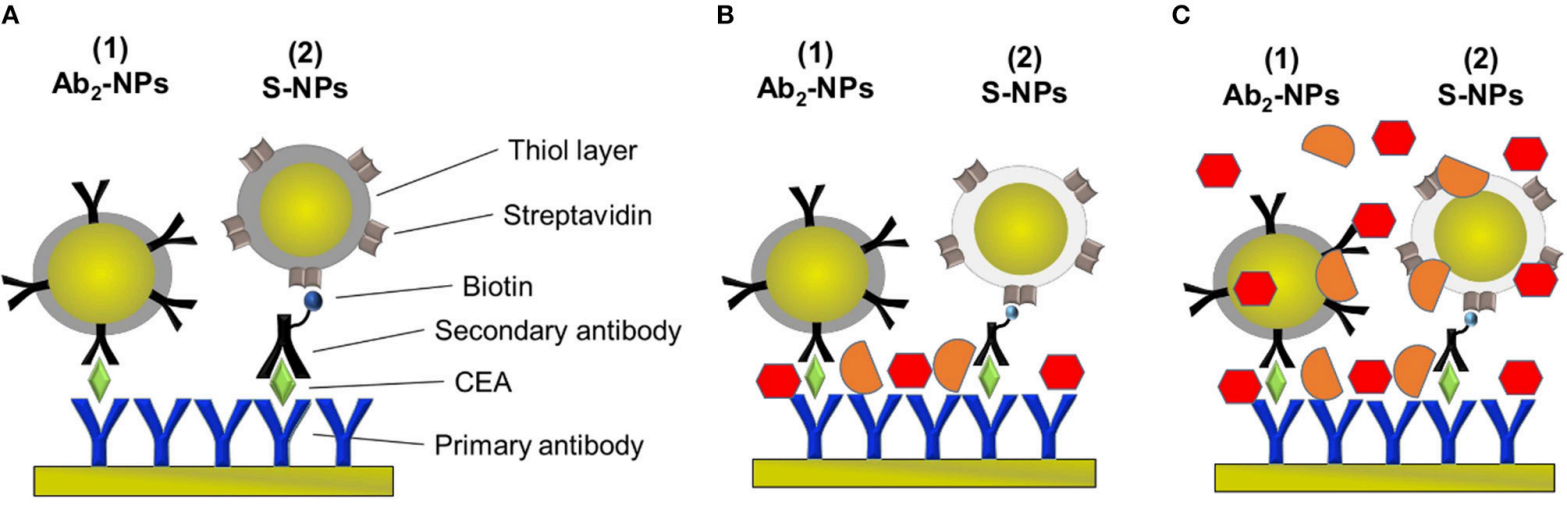
Schematic of the sandwich-assays using two types of functionalized NPs for the detection of CEA in the three different conditions studied in this paper. **(A)** Assay: Primary antibody immobilized on the sensor surface, incubated with EA in PBS_BSA_ and either NP functionalized with the secondary antibody specific for CEA (Ab_2_-NPs) or secondary antibody and NPs functionalized with streptavidin (S-NPs) binding to the biotinylated Ab_2_B. **(B)** Assay: Analogous to **(A)** except for that the sensor is exposed to plasma after CEA capture. **(C)** Assay: Analogous to **(A)** except for that the functionalized NPs are contained in blood plasma.

After the cancer marker detection, NP functionalized with the secondary antibody specific for CEA (Ab_2_-NPs) are used for the sensor response enhancement (Figure 2). Ab_2_-NPs are flowed on the surface overnight (20 μL/min, 0.33 nM), until the equilibrium phase, allowing recycling of the solution.

In the other approach, NPs functionalized with streptavidin (S-NPs) are bound to the secondary biotinylated antibody (Ab_2_B). Ab_2_B (10 μg/mL, 67 nM) is pumped through all channels for 15 min (20 μL/min) and then S-NPs are flowed overnight (20 μL/min, 0.33 nM), until the equilibrium stage, thereby recycling the sample (Figure 2). All SPR experiments reported in this work are performed at 25°C.

For both approaches, the reference channels are prepared with the same procedure described above, skipping the step of the CEA detection.

Three different ligand doses per NP (LDPN) are used for the surface functionalization of NPs in order to study the effect of NP-surface coverage on SPR sensor responses and Zpot values. The LDPN value corresponds to the ratio between the amount of ligand and the total number of NPs in solution. Zpot of the NPs and SPR performances in the bioassay are compared for different amounts of ligand useful for the functionalization of the NPs. The Bio-NP characteristics for biodetection are measured (i) first in buffer, and (ii) second in more complex matrix when the surface comes into contact with blood plasma. Finally, (iii) the same parameters are evaluated when the NPs are immersed in blood plasma and flowed overnight on the biosensor. For measurements mimicking sample analysis in blood plasma (ii), the procedure is the same as described above albeit with an additional injection of plasma for 5 min right after analyte injection. The sequential injection of target molecule and blood plasma is done as to maintain the density of target as similar as possible to experiments performed in buffer. In this way we obtain a surface similar to the one that we could have if the analyte would be detected directly from the blood plasma and we can directly see the binding of the target.

For measurements mimicking an *in vivo* analysis (iii), the procedure is the same as described above, albeit the Bio-NPs are diluted in 30% blood plasma in running buffer for the overnight detection.

## Results

### ζ-Potential Measurements

The ζ-potential (Zpot) of a colloidal solution is the electric potential, due to the net charge contained within the region, bounded by the slipping plane of the particles relative to that of the bulk fluid. It is related to the charge difference between the medium and the NP surface. In addition, the Zpot is directly related to the pH of the solution and it is an indicator of the colloidal stability.

**Figure 3:**
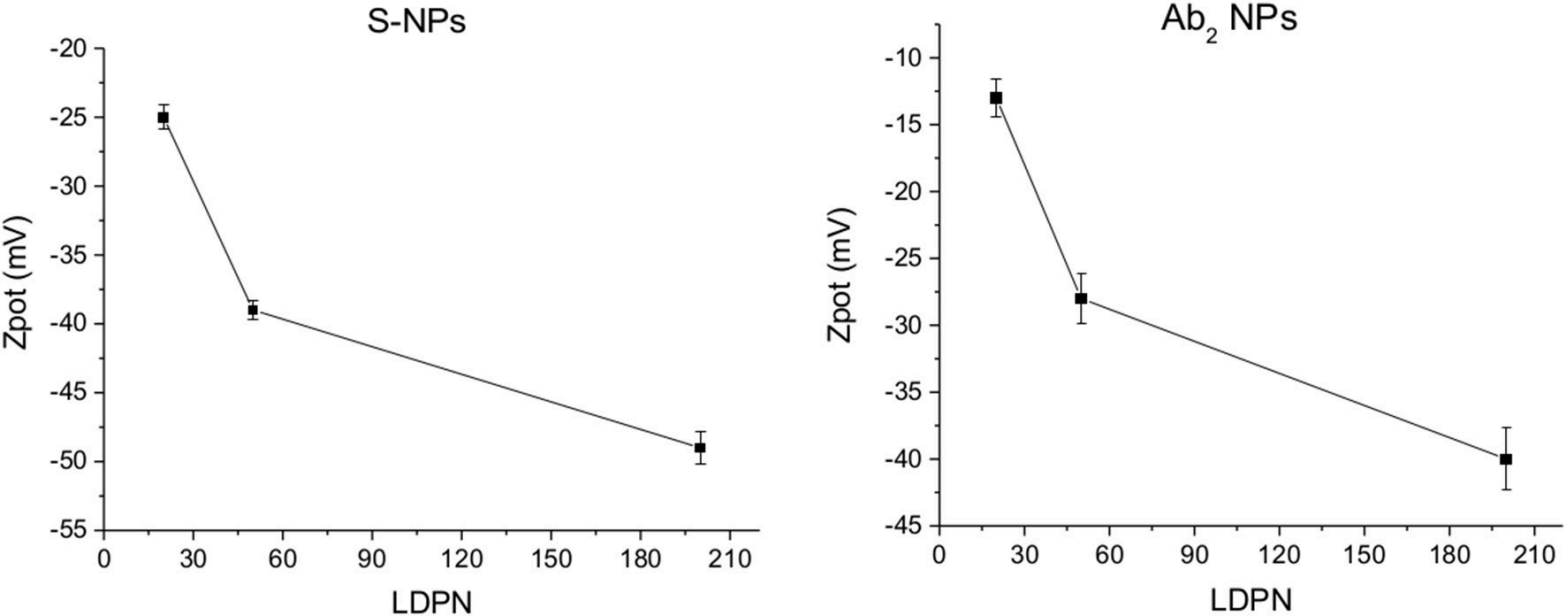
ζ-potential of Ab_2_-NPs **(left)** and S-NPs **(right)** as a function of LDPN. Measurements performed at pH 8 using a ZetaPals instrument.

The Zpot values of functionalized NPs are measured after functionalization with streptavidin (S) or CEA antibody (Ab_2_). Figure 3 shows Zpot values for the LDPNs. The measurements are performed around pH 8, above the isoelectric point of both ligands, thus the NPs overall charge is negative (the isoelectric point for streptavidin is known to be around 5, and the Zpot of the secondary antibody is measured to be negative). For each set of NPs, Zpot increased in absolute value with increasing LDPN, indicating an approaching saturation of the NP surfaces and an increased colloidal stability. The lowest values reached for the two batches of functionalized NPs differ from each other. We believe that this could be due to the difference in the isoelectric point of streptavidin, Ab_2_ and/or the difference in the amount of ligand bound.

### Characterization of NPs for *in vitro* Applications

**Figure 4:**
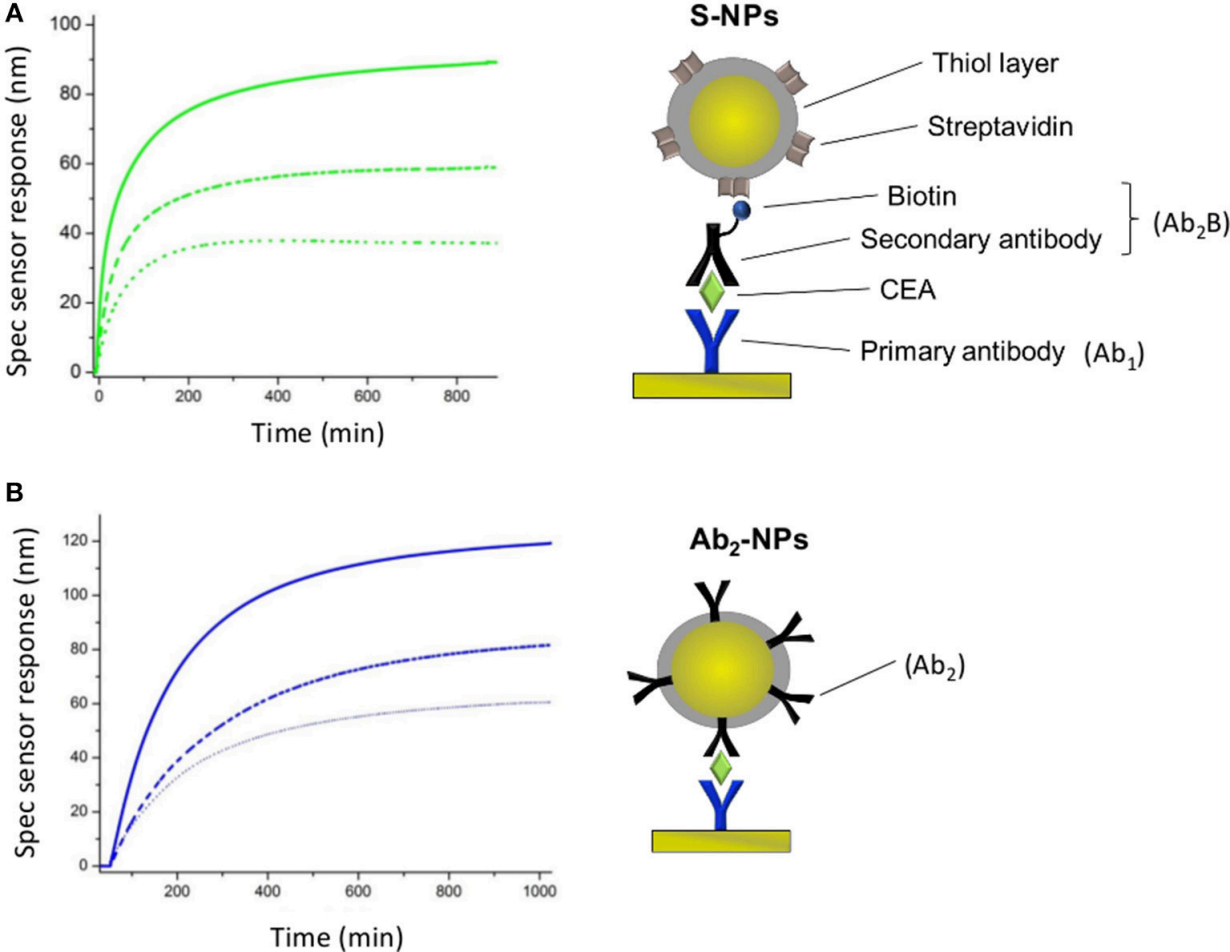
Specific SPR sensor responses for S-NPs **(A)** and Ab_2_-NPs **(B)** measured for three different LDPNs. The solid lines, dashed lines and dotted lines correspond to a LDPN of 200, 50, and 20, respectively. All the measurements were performed in PBS_BSA_. All the sensor response data are reference-compensated.

SPR sensor responses of both NPs are measured in the enhancing step of the CEA assays (Figure 2). Figure 4 show the specific SPR sensor responses (Spec) pertaining to the NP enhancing step, where Spec corresponds to the sensor response in the detection channel (Det) subtracted by the sensor response in the reference channel (Ref). For these experiments we set the contact time between NPs and the SPR biosensor surface long enough for the sensor response to reach a plateau, corresponding to equilibrium state (Figure 4).

For both types of Bio-NPs, the sensor response enhancement due to the NPs increases with LDPN. As a response of SPR biosensors is directly proportional to the amount of captured material, a higher sensor response indicates a higher amount of NPs attached to the sensing surface. Since the amount of target on the surface is kept constant in all the experiments, the difference in sensor response observed for different LDPN can be attributed to the different number of ligands on NPs that are available for the interaction with the ligands on the sensor surface.

**Figure 5:**
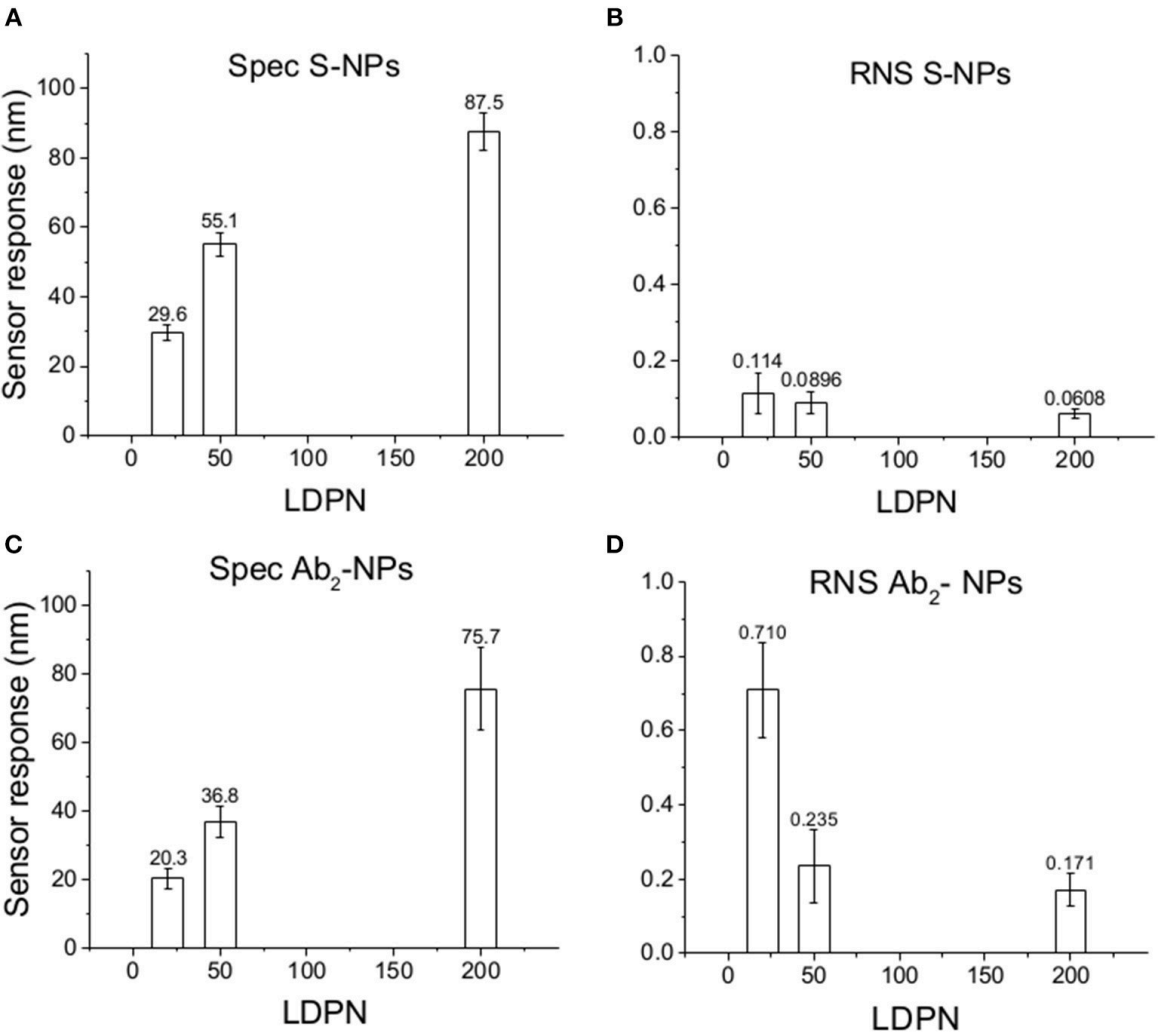
Specific SPR sensor responses to S-NPs **(A)** and Ab_2_-NPs **(C)** and relative non-specific SPR sensor response (RNS) to S-NPs **(B)** and Ab_2_-NPs **(D)** as a function of LDPN. The data were calculated as the mean values of SPR sensor responses at the equilibrium taken from at least 3 measurements. Measurements were performed in buffer (PBS_BSA_).

Figure 5 shows the relative weight of non-specific interactions on Spec, evaluated here by the relative non-specific SPR sensor responses (RNS), calculated here as Ref/(Det-Ref).

**Figure 6:**
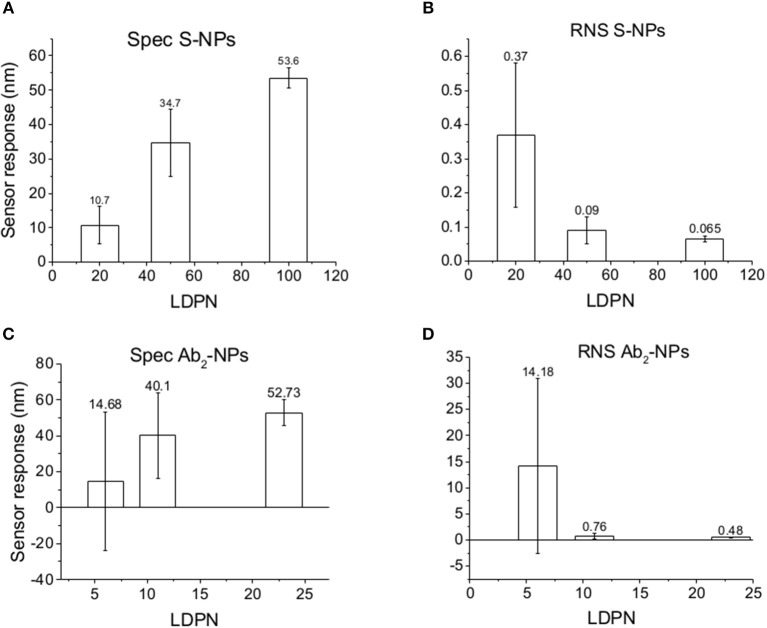
Specific SPR sensor responses to S-NPs **(A)** and Ab_2_-NPs **(C)** and relative non-specific SPR sensor response (RNS) to S-NPs **(B)** and Ab_2_-NPs **(D)** as a function of LDPN. The data were calculated as the mean values of SPR sensor responses at the equilibrium taken from at least 3 measurements. Measurements were performed in buffer (PBS_BSA_) after the biosensor surface was exposed to blood plasma.

Subsequently, the SPR sensor responses are evaluated under similar conditions as the previous set of data, but the surface is treated with blood plasma prior to the enhancing step (Figure 6). Those experiments are aimed to mimic the detection of the analyte immersed in such matrix.

The data in Figure 6 follow the same trend seen in buffer measurements (Figure 5), despite the more complex conditions: an increase in LDPN leads to an increase in the specific sensor response and a drastic decrease in non-specific interactions. It should also be noted that increases in LDPN lead to a decrease in the standard deviation of the measurements.

**Figure 7:**
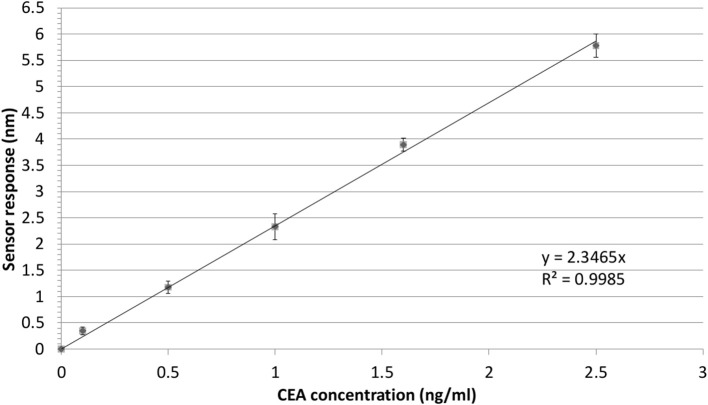
Detection of CEA in PBS_BSA_ employing sandwich assay with S-NPs: calibration curve. The SPR sensor response to the S-NPs (contact time with S-NPs solution: 10min) as a function of concertation of CEA. The error bars denote the standard deviation of the sensor response for each concentration. All the sensor response data are reference-compensated.

In order to illustrate the effect of the performance of the Bio-NPs on the limit of detection, the calibration curve for the detection of CEA using S-NPs is shown in Figure 7. The limit of detection was calculated as a concentration corresponding to 3 standard deviations of the blank sample and was determined to be 88.8 fM (17.8 pg/ml).

### Characterization of NPs Immersed in Blood Plasma

**Figure 8:**
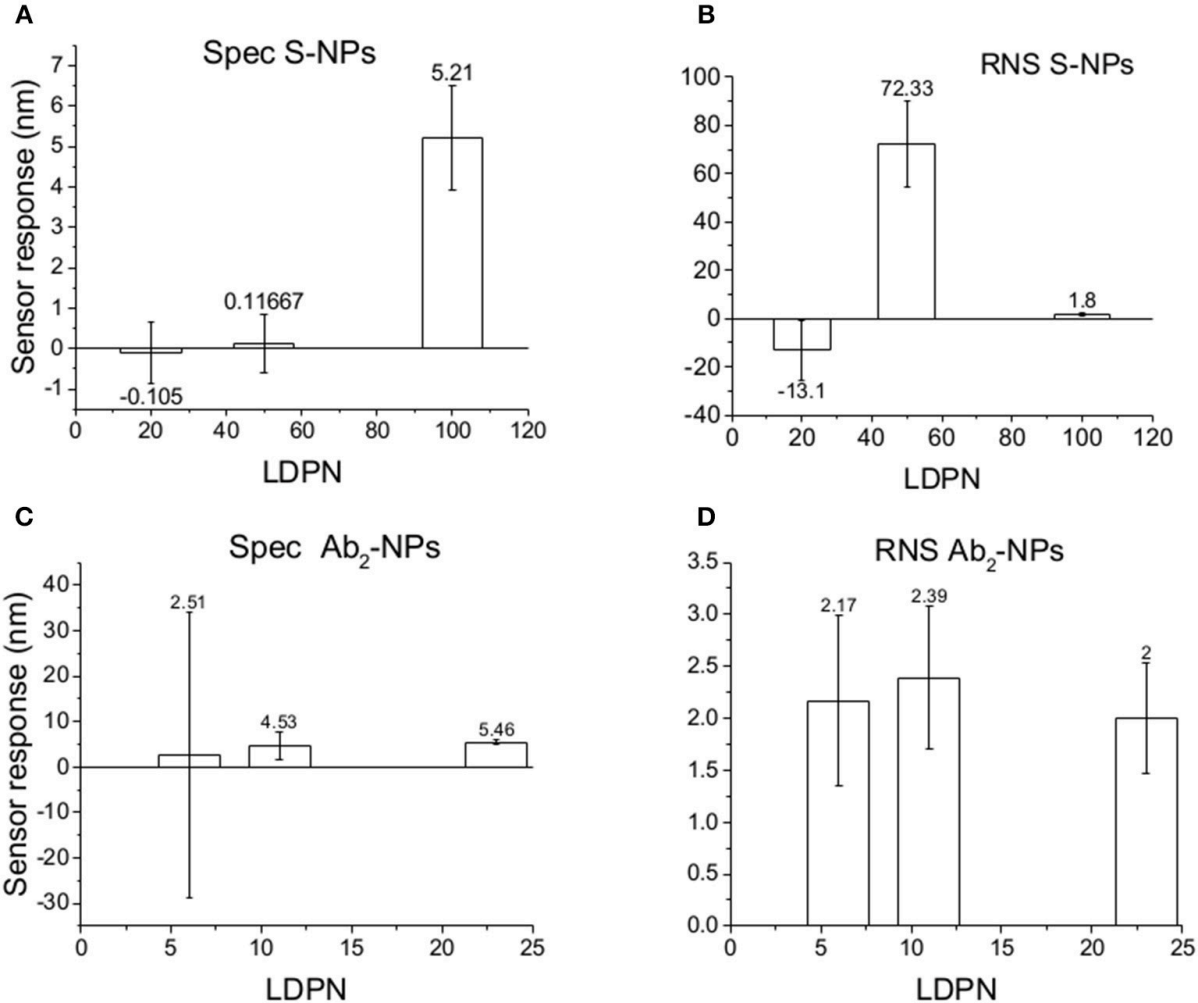
Specific SPR sensor responses to S-NPs **(A)** and Ab_2_-NPs **(C)** and relative non-specific SPR sensor response (RNS) to S-NPs **(B)** and Ab_2_-NPs **(D)** as a function of LDPN. The data were calculated as the mean values of SPR sensor responses at the equilibrium taken from at least 3 measurements. Measurements were performed in blood plasma diluted to 30% with PBS_BSA_.

We monitor the specific sensor response, the non-specific sensor response, and the reproducibility of the NP enhancement step over a range of 10 h, during which the NPs are constantly immersed in blood plasma. We obtain information about NP affinities, non-specific interactions, and robustness in such complex environmental conditions. Those experiments are aimed to mimic the stress caused by *in vivo* measurements on the NPs, or similarly, colorimetric sensing in a complex matrix. The results are shown in Figure 8.

For lowest LDPN the Ref is higher than Det, leading to negative RNS values. For the intermediate LDPN values, Ref is still very close to Spec. We observe a significant specific sensor response only for values corresponding to 100 LDPN, where we observe higher RNS values with respect to the previous detection in plasma, yet low enough to assure a successful detection.

## Discussion

The interaction between Bio-NPs and bio-functionalized surfaces in solution comprises a lot of dynamic forces and molecular-driven factors that, due to their complex interplay, are not describable with certainty (Nel et al., 2009). The main bio-physicochemical effects can be attributed both to the NP properties (shape, material, porosity, etc) and to the characteristics of the suspending media (pH, presence of salts or surfactants, etc). In a given medium the main forces acting at both interfaces are long range forces (Van der Waals), hydrophobic/hydrophilic interactions, steric forces, and electrostatic interactions with charges and double layers (Cosgrove, 2010). Those factors contribute to determine a complex system of effects that results in the behavior of NPs and are challenging control.

The complexity of such forces must be faced each time that NPs are functionalized with a ligand (e.g., the proteins on the Bio-NPs) interact with a bio-modified surface of a biosensor. An optimized functionalization should allow a specific interaction toward the ligand receptor, which is robust enough to assure high reproducibility and provides a minimal non–specific sensor response. Such properties dramatically influence the sensor response enhancement by NPs in biosensor measurements, especially in biological samples. Here we discuss the dependence of non-specific interactions, specificity and reproducibility of Bio-NPs on LDPN values, using a constant amount of the target molecule, on the basis of Zpot values and SPR measurements.

### ζ-Potential

The electrostatic field generated on the nanoparticle surface by the bio-modification with proteins (Zpot) depends on the amount of charges brought by the ligand. Accordingly, the results show that Zpot increases in absolute value when LDPN increases (Figure 3). This trend is consistent with the addition of an anionic ligand on negatively charged NPs previously reported in literature (Gamrad et al., 2014) when the number of negative ligands increases, the Zpot decreases and progressively the value becomes stable, signifying an approach to saturation of the NPs surface.

An efficient coverage of the gold creates a steric barrier that increases colloidal stability, keeping NPs far enough away from each other. Furthermore, the stronger the electrostatic surface potential, the higher the colloidal stability of the NPs, due to a balance of electrostatic forces. This stability is given by an energy barrier created by repulsive forces strong enough to counteract the Van der Waals and attraction forces, according to DLVO theory (Derjaguin et al., 1987). This energy is higher when the LDPN is increased due to the contribution of the peptides to the electrostatic field. We can suppose that this energy barrier not only maintains the NPs separated in solution, but also prevents the NPs from non-specifically sticking to the SPR sensor surface. Data show that when this energy barrier is sufficiently strong, i.e., high absolute values of Zpot, the energy of non-specific interactions is less able to destabilize NPs from the solution and the non-specific interactions are minimized.

### Non-specific Sensor Response

The SPR sensor response for the reference channel (Ref) reflects the intensity of all non-specific interactions of the Bio-NPs during the assay and is not driven by the specific recognition between ligand and receptor. The sensitivity of the NP-enhanced sensor response, in terms of limit of detection, is dependent on the behavior of the NPs in both reference and detection channels. For this reason, we consider the relative non-specific sensor response (RNS), which describes the intensity of the non-specific sensor response relative to the specific one [reference/(detection-reference)]. In other words, it expresses the weight of the non-specific interactions in the detection with respect to the specific interactions.

When comparing Zpot and RNS in buffer we can see that an increase of absolute value of Zpot leads to both a decrease in non-specific interactions as well as a higher affinity (higher Spec), resulting finally in drastically lower RNS (Figure 5). This trend is shown to be the same for all the NPs studied in buffer and also when the biosensor surface is treated with plasma. The contact with plasma changes the characteristics of the biosensor surface, so that the NPs have to face a more complex environment during target recognition. The target-NP interactions happen regardless, demonstrating that NPs are able to overcome the layer of molecules deposited after plasma and finally reach the CEA (Figure 6). It should be noted that Ab_2_-NPs exhibit RNS values that are higher than those provided by S-NPs. The difference in the performance of the two types of Bio-NPs is more pronounced at low LDPN values. For the lowest LDPN, RNS provided by Ab_2_-NPs is about 5–6 times higher than that of S-NPs, both in buffer and for the sensor surface exposed to blood plasma. We believe that this is due to the nature of the antibody being more complex than streptavidin.

When NPs are immersed in blood plasma for several hours, their sensing characteristics are significantly affected. It is known that in complex matrixes the biomaterials tend to create a layer on NP surface [corona (Lundqvist et al., 2011)] that is responsible for the NP fate. Even if this does not compromise the colloidal stability in solution, this effect could create steric problems or energy barriers, stronger than the affinity interaction, which either impede bio-recognition or enhance the non-specific interactions. Although NPs are never aggregated in the stock solution during these experiments, results differed from the previous two experiment types. RNS values using Ab_2_-NPs are quite high compared with the detection channel and not highly influenced by the LDPN (Figure 8). This suggests that non-specific interactions are mainly driven from the protein corona, where the resulting NP binding is unaffected by the inner layers. Data from the S-NPs show negative RNS for the lowest LDPN, indicating that Ref is even higher than Spec, and for middle LDPN, RNS is extremely high (detection and reference are very similar). Finally, RNS for the S-NPs at the highest LDPN is 1.8, which is higher than the value obtained in the measurements in buffer, yet still indicating the existence of a significant specific detection.

### Specific Sensor Response

The specific sensor response, Spec, is related to the NPs affinity for the target molecule, hence also to their binding rate in the assay as well as their equilibrium density on the biosensor surface. When comparing different ligands, one must take into account the characteristics of the ligand molecule themselves (i.e., binding characteristics when not NP-bound). In our case the streptavidin-biotin interaction is stronger than the antibody-ligand reaction, thus we observed differences in binding rates even when those molecules are attached to NPs. Spec also depends on their density on the NPs surface. It has been shown by Soukka et al. (2001) and Li et al. (2014) that efficiently binding NPs are obtained when the ligand number surpasses a certain threshold required for multivalent binding, suggesting that avidity (affinity to multiple receptors) could be an important factor for fast and efficient binding. For both batches and the assay in buffer, Spec at equilibrium increases with LDPN, following the trend of the Zpot (Figure 5).

A similar situation occurs when the surface is treated with plasma: the intensity of Spec is lower compared to buffer, probably because blood plasma covers some of the analyte on the biosensor surface (Figure 6). When NPs are immersed in plasma, the differences of Zpot inside every batch generate much larger differences in experimental performance. For low Zpot (and LDPN) Spec is remarkably low, because Ref is higher than Spec and noticeably higher than previous values. For high LDPN, Spec is significantly different from the blank, suggesting that those highly functionalized NPs can maintain their specific functionality even after an overnight immersion in blood plasma (Figure 8). When comparing the two types of Bio-NPs, one can conclude that their performance expressed in terms of Spec is comparable in all the investigated media for the highest LDPN values. When LDPN is reduced, the performance of Ab_2_-NPs in 30% plasma becomes less reproducible; however, in other tested media, it remains similar to the performance of S-NPs.

### Reproducibility

Having an overall look to SPR sensor responses in buffer solution, we can notice that the dispersion of the data around the average value is very similar for different LDPN. On the other hand, when we move to sensor response enhancement on plasma-treated surfaces, we can see that measurements are not reproducible for low LDPN, especially for the RNS regarding antibody coated NPs. For highest LDPN, both Spec and RNS show high reproducibility based on low standard deviation. This trend is more evident again when it comes to NPs immersed in plasma overnight, where a very good reproducibility is maintained for high LDPN, despite the complex conditions. Again, the surface design of the NPs dictates the quality of the detection, which is in agreement with Zpot value trends.

## Conclusions

The surface design of the Bio-NPs is crucial for clinical detections which involve functional nanoparticles, from *in vitro* measurements to *in vivo* applications. We show that, for the same amount of target molecule and when just tuning the properties of the Bio-NPs surface, the NP sensor response enhancement can range from 0 to 800 times the sensor response of the target analyte. With a quite simple Zpot measurement, it is possible to obtain useful information for improving the performance of Bio-NPs, aiming toward a highly specific interaction with the target molecule, a very low non-specific sensor response, and excellent reproducibility. These improvements can be successfully obtained for both conditions (in buffer and in blood plasma), confirming that our findings can be of great importance for clinical applications.

Furthermore, we also open the prospective to use the SPR biosensors as a tool for an optimized design of Bio-NPs for *in vivo* application. It is interesting to notice that often *in vivo* experiments lack possibility of control or reference, and it is challenging to distinguish between specific and non-specific interactions. With SPR, the non-specific interactions of Bio-NP immersed in blood plasma are constantly monitored in parallel with the specific interactions, thus giving a clear overview of the behavior of Bio-NP properties under complex conditions in real time.

## Author Contributions

ME made substantial contributions to conception and design, acquisition of data, analysis, and interpretation of data, drafting the article. TŠ and XC contributed to the acquisition, analysis and interpretation of the data, and participated in revising the article. JH contributed substantially to conception and design, analysis and interpretation of data, drafting the article and in revising the article critically for important intellectual content. All authors give final approval of the version to be submitted.

### Conflict of Interest Statement

The authors declare that the research was conducted in the absence of any commercial or financial relationships that could be construed as a potential conflict of interest.
